# The complete chloroplast genome sequence of purple mullein (*Verbascum phoeniceum* L.)

**DOI:** 10.1080/23802359.2020.1715880

**Published:** 2020-01-24

**Authors:** Yuqian Bi, Pan Deng, Luxian Liu

**Affiliations:** Key laboratory of Plant Stress Biology, School of Life Sciences, Henan University, Kaifeng, China

**Keywords:** Purple mullein, chloroplast genome, phylogeny inference

## Abstract

*Verbascum phoeniceum*, known as purple mullein or temptress purple, is a species native to Central Europe, Central Asia, and Western China. In the present study, the chloroplast (cp) genome of *V. phoeniceum* was assembled using genome skimming sequencing. The cp genome of *V. phoeniceum* is 153,348 bp in length comprising two copies of inverted regions (IR, 25,430 bp) separated by the large single-copy (LSC, 84,601 bp) and small single copy (SSC, 17,887 bp) regions. It encodes 114 unique genes, consisting of 80 protein-coding genes, 30 tRNA genes, and 4 rRNA genes, with 20 duplicated genes in the IR regions. Phylogenetic analysis indicates that *V. phoeniceum* exhibits a closer relationship with *Scrophularia* rather than *Buddleja*.

The Scrophulariaceae are a family of flowering plants, commonly known as the figwort family and the plants are annual and perennial herbs as well as one genus of shrubs (Fischer 2004). The family includes 62 genera and about 1830 known species (Christenhusz and Byng 2016) and the phylogenetic relationships of species in Scrophulariaceae remain one of the most problematic topics in angiosperm systematics (Judd and Olmstead 2004). Recently, the cp genome has been used as an effective tool for phylogenetic studies (Liu et al. 2017). However, cp genomes reported in this family only involved *Scrophularia* L. (Xu et al. 2019) and *Buddleja* L. (Ge et al. 2018). In this study, the first complete cp genome from *Verbascum* L. (*V. phoeniceum*) was sequenced using genome skimming data. The genome sequence was registered into GenBank with the accession number MN893301.

One individual of *V. phoeniceum* was collected from Xinjiang (China; 81°10′32.15″E, 44°27′12.23″N) and a voucher specimen (*Pan Li LP173548*) was deposited at the Herbarium of Zhejiang University (HZU). Total genomic DNA was extracted from silica-dried leaves using Plant DNAzol Reagent (LifeFeng, Shanghai) according to the manufacturer’s protocol. High-quality DNA was sheared and the paired-end library (≤800 bp) was sequenced on an Illumina HiSeq X10 at Beijing Genomics Institute (BGI, Wuhan, China). The raw data were screened by quality with Phred score <30 and assembled into contigs using the CLC Genomic Workbench (CLC Inc. Aarhus, Denmark). The complete cp genome of *V. phoeniceum* was constructed with *Scrophularia henryi* (GenBank accession number: MF861203) as a reference and annotated using the software Geneious R11 (Biomatters, Auckland, New Zealand) following description in Liu et al. (2018). Phylogenetic tree for 28 whole cp genome sequences of Lamiales was constructed using Maximum Likelihood (ML) method implemented in RAxML-HPC v8.1.11 on the CIPRES cluster (Miller et al., 2010) with *Tiquilia plicata* as outgroup.

The complete cp genome of *V. phoeniceum* is 153,348 bp in length, consisting of 84,601 bp LSC (large single-copy) region, a 17,887 bp SSC (small single-copy) region, and a pair of 25,430 bp IR (inverted repeat) regions. In total, 114 unique genes were annotated including 80 protein-coding genes, 30 tRNA genes, and 4 rRNA genes, additionally with 20 duplicated genes in the IR regions. Six tRNA genes and eight protein-coding genes contain a single intron, and three genes (*rps*12, *clp*P, and *ycf*3) contain two introns. The overall GC content of the total length, LSC, SSC, and IR regions is 38.0, 36.1, 32.3, and 43.2%, respectively. The constructed phylogeny revealed that *V. phoeniceum* and four *Scrophularia* species formed a strongly supported clade, which in turn was sister to *Buddleja* (Figure 1).

**Figure 1.**
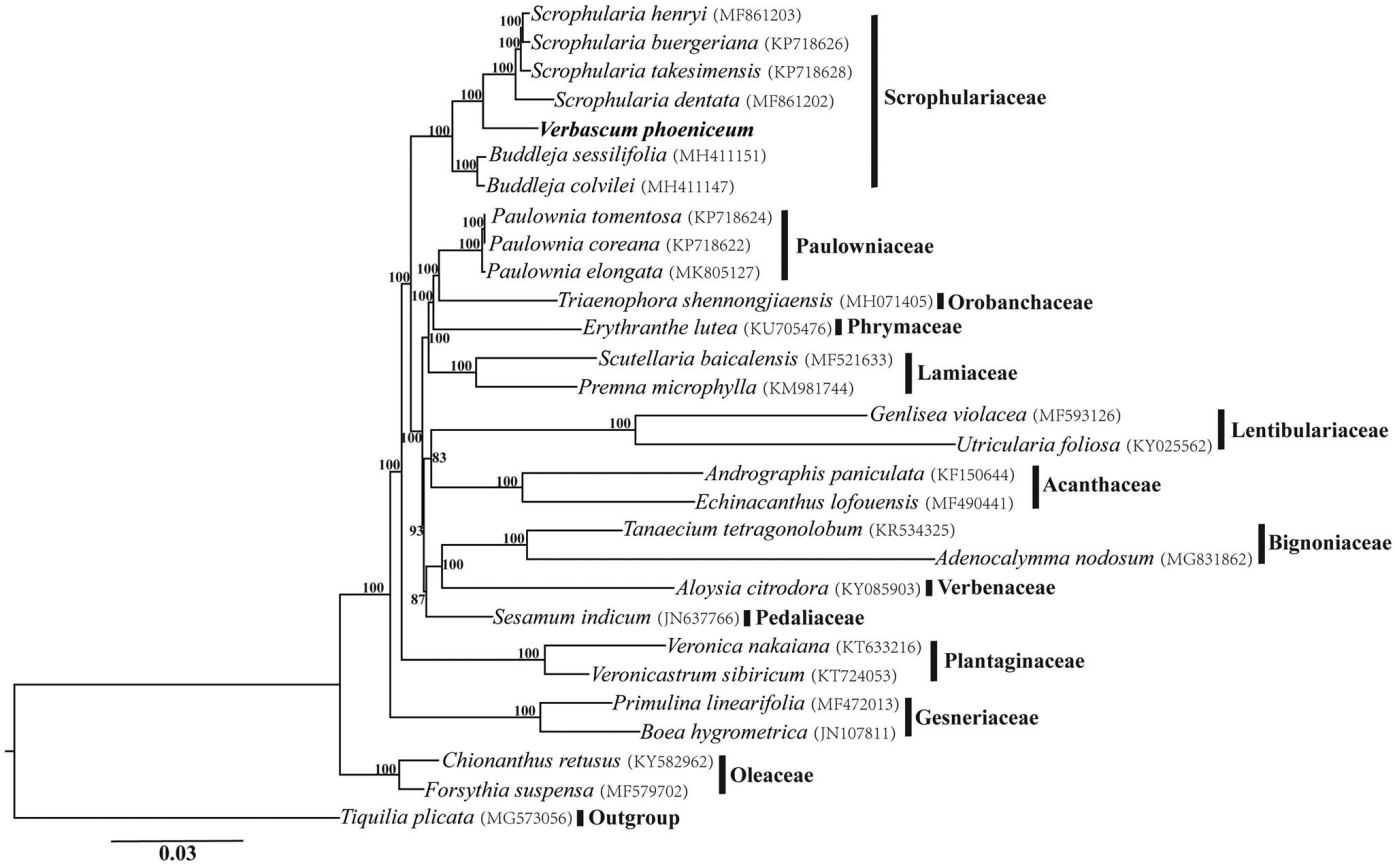
Phylogenetic relationships of Lamiales inferred based on whole chloroplast genome sequences. Numbers above the branches represent bootstrap values from maximum-likelihood analyses.
